# Does small bite closure reduce the incidence of incisional hernia compared to standard mass closure for midline laparotomy?

**DOI:** 10.1016/j.amsu.2022.104485

**Published:** 2022-08-24

**Authors:** Kareemaldin Elsamani, Ahmed Abdel Rahim, Safaa Hamid

**Affiliations:** a Department of General Surgery, University of Bahri, Khartoum, Sudan; b Vascular Surgery Registrar, Aswan University Hospitals, Egypt; c General Surgery Registrar, Sudanese Medical Specialization Board, Sudan

## Abstract

A best evidence topic was constructed using a defined protocol. The three-part question addressed was: in closure of midline laparotomy, which technique had lower incidence of incisional hernia: small bite closure or mass closure? The best evidence demonstrated that small bite technique has lower incidence of hernia.

## Highlights

• Small bite closure technique has lower incidence rates of incisional hernia compared to conventional mass closure.
• In Elective midline laparotomies, small bite closure should be the standard technique.
• A maximum of 5 mm space from the edge of the sheath and between stitches was superior to the 10 mm distance in mass closure.

## 1 Introduction

Best evidence topic is constructed using a well-defined protocol described by the international journal of surgery [1]. This format was used because a preliminary literature search suggested that the available evidence is insufficient to perform a meaningful meta-analysis. A BET provides evidence-based answers to common clinical questions using a systematic approach of reviewing the literature.

## 2 Clinical scenario

A general surgery trainee was discussing the technique of abdominal wall closure during an elective laparotomy for right hemicolectomy with his consultant and suggested to perform a small bite closure instead of mass closure, the consultant asked for evidence to prove if this technique is better, specifically in reducing incidence of incisional hernia.

## 3 Three-part question

[In closure of midline laparotomy] [does small stitch technique compared to mass closure of abdominal wall] [has lower incidence of incisional hernia]?

## 4 Search strategy

• Medline ® 1946 to July 2022 and Embase 1974 to July 2022 using Ovid interface:

[laparotomy OR Midline laparotomy OR Midline incision OR abdominal incision] AND [incisional hernia OR IVH OR hernia OR hernia incidence] AND [Small bite closure OR small bites OR short stitch OR short stitch OR small bite technique OR large bite technique OR large stitch OR big bite OR long stitch OR Mass closure].

• Medline ® using PubMed interface:

[Laparotomy OR midline laparotomy OR midline incision OR Laparotomy] AND [small bite closure OR small stitch OR small bites technique OR short bite OR small bite OR short stitch OR mass closure OR large bite OR big bite OR long stitch] AND [hernia OR incisional hernia OR IVH OR hernia incidence].

The results were limited to English articles and human studies.

• Inclusion criteria: all original articles that review the post-operative outcomes in patients who underwent elective laparotomy.
• Exclusion criteria: studies in children, case reports, letters to the editor, conference abstracts and systematic reviews, and meta-analysis.

## 5 Search outcomes

The total number of studies identified initially after removal of duplicates was 220. Of these, 202 were excluded based in abstracts and titles, in addition to studies where prophylactic mesh was used for closure. The final 18 studies were requested and fully assessed by reviewing the full text, and further 12 studies were excluded after deemed unsuitable. This resulted in 6 studies (4 randomized controlled trials and 2 retrospective cohorts) included in generating the best evidence to answer this question.

## 6 Results

See Table 1.

**Table 1:** Result.

Author, year of publication, journal name and country	Study type and level of evidence	Patient group (SB = small bite LB = large bite)	Outcomes & follow up	Key results	comments
*Deerenberg* et al. [2] 2015 Lancet The Netherlands	Prospective randomized controlled trial level II	A total of **545** patients: **(SB) group: 268** **(LB) group: 277**	Primary outcome: Incidence of incisional hernia at follow-up period (12 months)	**SB Group: 35 (13%)** **LB Group: 57 (21%)** **(P = 0.0220)** ***Difference is statistically significant***	- multi centre, double-blinded study -large sample size - Relatively short period of follow up
*Fortelny* et al. [3] 2022 The British Journal of Surgery Austria	prospective randomized controlled trial level II	A total of **414** patients: **(SB) group: 210** **(LB) group: 204**	Primary outcome: Incidence of incisional hernia at follow-up period (12 months)	**SB Group: 7 (3.3%)** **LB Group: 13 (6.4%)** **(P = 0.173)** ***Difference is not statistically significant***	- multi centre, double-blinded study -large sample size - Relatively short period of follow up
*Millbourn* et al. [4] 2009 Archives of Surgery Sweden	prospective randomized controlled trial level II	A total of **522** patients: **(SB) group: 250** **(LB) group: 272**	Primary outcome: Incidence of incisional hernia at follow-up period (12 months)	**SB Group: 14 (5.6%)** **LB Group: 49 (18%)** **(P<0.001)** ***Difference is statistically significant***	-Single centre -Large sample size -Relatively short period of follow up
*Harlaar* et al. [5] 2017 British Journal of Surgery Netherlands	prospective randomized controlled trial level II	A total of **219** patients: **(SB) group: 113** **(LB) group: 106**	Primary outcome: Incidence of incisional hernia at follow-up period (12 months)	**SB Group: 22 (19.5%)** **LB Group: 38 (35.8%)** **(P = 0.007)** ***Difference is statistically significant***	- Single centre - Large sample size - Relatively short period of follow up
*Söderbäck* et al. [6] 2022 Langenbeck's Archives of Surgery Sweden	Retrospective Cohort Level III	A total of **1120** patients: **(SB) group: 518** **(LB) group: 602**	Primary outcome: Incidence of incisional hernia at follow-up period (36 months)	**SB Group: 21 (4.3%)** **LB Group: 32 (5.1%)** **(P = 0.52)** ***Difference is not*** ***Statistically significant***	- Single centre - Retrospective, - Large sample size - Long follow up
*De Vries* et al. [7] 2019 Hernia Journal The Netherlans	Retrospective Cohort Level III	A total of **327** patients: **(SB) group: 136** **(LB) group: 191**	Primary outcome: Incidence of incisional hernia at follow-up period (16 months)	**SB Group: 10 (7%)** **LB Group: 27 (14%)** **(P = 0.08)** ***Difference is not*** ***Statistically significant***	- Single centre - Retrospective, - Large sample size - Long follow up

## 7 Discussion

Incisional hernia is one of the late complications of midline laparotomy incision that carries significant morbidity to patients and can be very challenging for surgeons to manage. The incidence of incisional hernia following midline laparotomy is 5%–41% and the wide variation between studies, is owed mainly to the difference in length of follow up [8].

Over the last decade there has been intensive studies and trials on the prevention and reduction of incisional hernias and the main focus was on the technique as it is the only independent factor that is controlled by the surgeon. Many techniques have been explored, including use of prophylactic mesh [9], distance of stitches from sheath edge and each other (5 mm instead of the conventional 10 mm in mass closure), and different types of suture materials and length [10].

The goal of the review was to answer the question posed at the start of the article; whether small bites can reduce the incidence of incisional hernia following midline laparotomy when compared to the conventional mass closure technique, with the latter being the regular practice of most of today's surgeons for several years if not for their entire career which makes the transition even more difficult if another technique is proven to be superior [11,12]. Nevertheless, we think by generating high-quality evidence of the small bite/small stitch technique in the form of best evidence topic, might help in adoption of this technique by more units.

A total of 5 high-quality studies were used to generate this review, 4 RCTs, 3 studies showed a statistically significant difference in the incidence of incisional hernia in favour of the small bite technique, but in all studies the incidence was higher in the large bite group. All studies have large sample size, and 3 were double-blinded and multicentric, while the other 3 studies [4,5] were single centre and not blinded. Follow up period of 12 months amongst most the studies was relatively short, given that incisional hernia can develop years after primary operation [13]. Although *Söderbäck* et al. [6]*& De Vries* et al. [7] were retrospective cohorts, they both had a study and control groups and a relatively large cohort of patients with a mean follow up 0f 16 and 36 months respectively.

## 8 Clinical bottom line

Based on the findings from the studies above, the small bite technique of midline laparotomy incisions is superior to the conventional large bite/mass closure in reducing the incidence of incisional hernia.

## 9 Limitations of the review

1. Short follow up period in most of the studies
2. Some studies were single centric.

## Ethical approval

Not applicaple.

## Source of funding

None.

## Author contribution

Kareemaldin Elsamani (KE): performed the literature search and wrote the paper.

Ahmed Abdel Rahim (AA): helped in search and writing the paper.

Safaa Hamid (SH): Helped in editing.

## Declaration of competing interest

None.

## Consent

Ethics committee approval was not required as the study was review of previously done studies.

## Registration of research studies

1. Name of the registry:
2. Unique Identifying number or registration ID:
3. Hyperlink to your specific registration (must be publicly accessible and will be checked):

## Guarantor

Kareemaldin Elsamani
